# Prediction of disulfide bond engineering sites using a machine learning method

**DOI:** 10.1038/s41598-020-67230-z

**Published:** 2020-06-25

**Authors:** Xiang Gao, Xiaoqun Dong, Xuanxuan Li, Zhijie Liu, Haiguang Liu

**Affiliations:** 1 Complex Systems Division, Beijing Computational Science Research Center, 8 E Xibeiwang Rd, Haidian, Beijing, 100193 People’s Republic of China; 2 School of Software Engineering, University of Science and Technology China, Suzhou, Jiang Su 215123 People’s Republic of China; 3 Department of engineering physics, Tsinghua University, Haidian, Beijing, 100084 People’s Republic of China; 4 iHuman Institute, ShanghaiTech University, 393 Middle Huaxia Rd, Pudong, Shanghai, 201210 People’s Republic of China

**Keywords:** Computational biophysics, Machine learning

## Abstract

Disulfide bonds are covalently bonded sulfur atoms from cysteine pairs in protein structures. Due to the importance of disulfide bonds in protein folding and structural stability, artificial disulfide bonds are often engineered by cysteine mutation to enhance protein structural stability. To facilitate the experimental design, we implemented a method based on neural networks to predict amino acid pairs for cysteine mutations to form engineered disulfide bonds. The designed neural network was trained with high-resolution structures curated from the Protein Data Bank. The testing results reveal that the proposed method recognizes 99% of natural disulfide bonds. In the test with engineered disulfide bonds, the algorithm achieves similar accuracy levels with other state-of-the-art algorithms in published dataset and better performance for two comprehensively studied proteins with 70% accuracy, demonstrating potential applications in protein engineering. The neural network framework allows exploiting the full features in distance space, and therefore improves accuracy of the disulfide bond engineering site prediction. The source code and a web server are available at http://liulab.csrc.ac.cn/ssbondpre.

## Introduction

Disulfide bonds play critical roles in protein folding, stability, and functions^1^. Stability of the target protein could be reduced if native disulfide bonds were removed^2^. On the other hand, creating new disulfide bonds in protein molecules by engineering may improve structural stability or rigidity^3,4^. De novo functions can be developed after introducing disulfide bonds into the proper positions in protein molecules. In consideration of structure determinations at high resolution either by using crystallography or cryogenic electron microscopy single particle imaging methods, structures with enhanced thermostability are often essential to keep molecules in a single conformation.

Disulfide bond predictions can be classified into two categories, depending on the available information and the goals. Sequence based disulfide bond prediction methods are mainly applied to wild type proteins, with the goal of predicting the bonding states of cysteine residues in wild type proteins^5,6^. It has been shown that successful prediction of naturally occurring disulfide bonds can improve the accuracy of 3D structure prediction^7^. On the other hand, structure-based disulfide bond prediction has a different focus, aiming to predict the likelihood of forming engineered disulfide bonds by mutating other types of amino acids to cysteine. The former facilitates protein structure predictions, and the latter is applied more frequently in protein engineering. In this work, we tackle the latter problem and develop a new method for the prediction of mutation sites to form engineered disulfide bonds using a machine learning approach. The geometry arrangement of the candidate bonding residues, either wild type cysteine or cysteine mutated from other amino acids, is the major consideration in disulfide bond engineering. For example, the MODIP program utilizes the knowledge of stereochemical information to predict the point mutations that can lead to engineered disulfide bonds. In the revised MODIP, a set of 538 high-resolution structures (of which 172 have disulfide bonds) were selected from the protein databank to study the geometry arrangement of disulfide bonded cysteine residues. Specifically, the geometry information was represented using the distances between the alpha-carbons and the beta-carbons of the bonded cysteine residues, along with the three torsion angles around the disulfide bonds (χ^1^,χ^ss^,χ^1’^)^8,9^. A similar approach was implemented in the Disulfide by Design programs (DbD and DbD2), which include the angles formed by the atoms Cα-Cβ-Sγ for each cysteine^10,11^. By optimizing the geometry, the DbD programs can be used to predict the positions of the mutated cysteine atoms. Salam *et al*. applied both empirical knowledge about the disulfide bond geometry and physical interactions between neighboring atoms to predict disulfide bonds in mutant proteins based on wild type structures^12^. A disulfide bond prediction algorithm that aims to improve crystal quality has been developed to locate the potential disulfide bonds that lead to lower entropy of the protein molecules by using geometry restraints and support vector machine method^13^.

Although the statistical knowledge about geometry is widely applied in engineering disulfide bonds in proteins, the performance is limited by the artificially selected features, which are often representative of simple analytical relations between atoms (i.e., bond distances, bond angles, or torsion angles). In recent years, the machine learning approach has made tremendous advances, mainly due to the development of high-performance computing technology and the accumulation of high quality experimental data. Using the framework of machine learning, one can go beyond the existing knowledge that may limits the feature design and selection, by allowing the machine/algorithm to exploit the full feature space and select the significant features by training. In this work, we designed a neural network model to learn the structure features that are associated with disulfide bonds. The neural network training is fully based on the coordinates of atoms that are involved with the potential disulfide bonds. The training dataset was extracted from a set of PDB structures after removing sequence/structure redundancy. The testing results showed that the algorithm has excellent performance in recognizing naturally occurring disulfide bonds. Furthermore, the algorithm was tested with engineered disulfide bonds and showed good performance. The algorithm is available as a standalone program named *SSbondPre* and a web-server at http://liulab.csrc.ac.cn/ssbondpre.

## Methods

### Training and testing datasets

**Figure 1:**
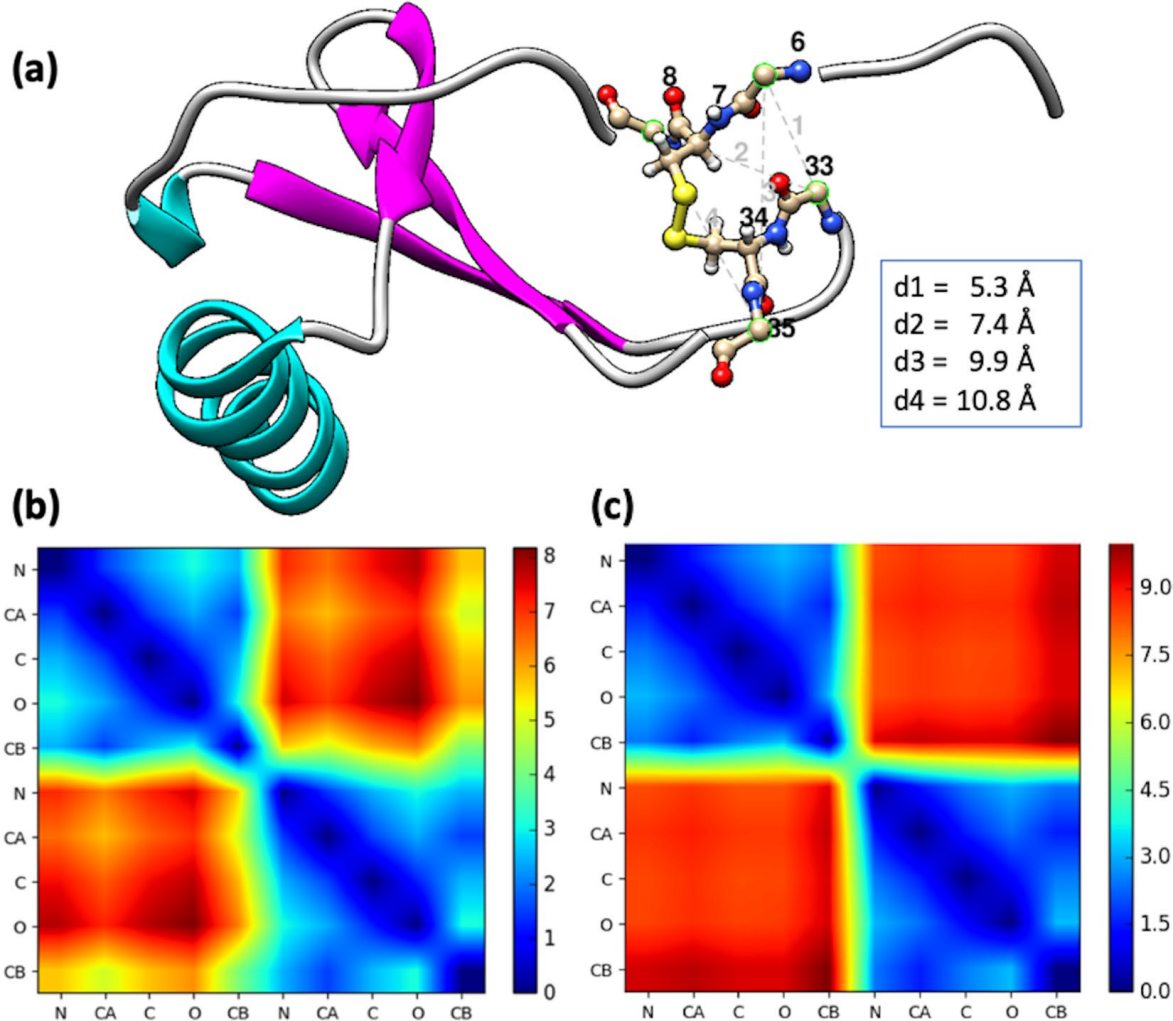
The disulfide bond and training dataset derivation. (**a**) A disulfide bond observed in a protein (PDB ID: 1IL8), and a negative sample is generated by finding the nearest neighbors between the adjacent residues of the cysteine residues. (**b**) The average distances between atoms of disulfide bonded cysteine amino acids in a heatmap representation. (**c**) The average distances between atoms of amino acid pairs in the negative sample set.

To train a classification neural network, a labelled dataset composed of two classes of data is required. Here, we refer to the bonded cysteines observed in protein structures as positive samples. From a subset of structures downloaded from the Protein Data Bank, after removing the redundancy using NCBI VAST (the vector alignment search tool) programs^14^, the dataset was derived for training and testing. The PDB and chain ID’s were retrieved from the VAST website (https://www.ncbi.nlm.nih.gov/Structure/VAST/nrpdb.html). The non-redundant structure dataset is composed of 14,647 proteins with the p-value cutoff at 10^−7^, the information obtained from the VAST server were used to download the structure files from the Protein Data Bank. In the next step, using the ‘SSBOND’ tag in the PDB files, 12,496 disulfide bonds were extracted. For the negative samples, the free cysteines that do not form disulfide bonds were not used, because the proposed algorithm is for disulfide bond engineering based on structural information, without referring to protein sequence information in this study. For network model training and disulfide bond prediction, only the backbone and C_β_ atoms from each amino acid were used (see section 2.2 for details), the protein sequence information is neglected, therefore, the method is not sensitive to protein sequence. We applied the following procedure to compile the negative sample set (see Fig. 1a): (1) identify two sequentially neighboring amino acids for bonded cysteines (C_i_,C_j_), resulting in four neighboring amino acids for each disulfide bond (X_i-1_, X_i+1_; X_j-1_, X_j+1_); (2) compute the Cα distances between the four crossing pairs of amino acids (X_i-1_X_j-1_, X_i-1_X_j+1_, X_i+1_X_j-1_, X_i+1_X_j+1_); and (3) choose the pair with the shortest Cα-Cα distance as the negative sample derived from the corresponding naturally occurring disulfide bond. For example, the SSBOND indicate that the Cys-7 and Cys-34 form a disulfide bond (Fig. 1a); then the negative sample can potentially be one of the following paired residues (the numbers are the residue ID’s): 6–33, 6–35, 8–33, 8–35. After comparing the distances between the Cα atoms for each pair of residues, the residues 6 and 33 were selected as the negative dataset in this case. The same procedures were carried out for the selected VAST dataset, and 12,496 negative samples were generated. Out of 24,992 samples (including both positive and negative samples), 18,992 samples were used in the training set to optimize the neural network parameters, and 6,000 samples were used as testing samples. Considering that the proposed algorithm solely depends on the structure (not sequence), the structurally dissimilar dataset generated by VAST method was used as the main dataset. To rule out the potential bias due to the selected dataset, we carried out another analysis using the dataset with less than 40% sequence identify from the PISCES webserver^15^. The structures with resolutions better than 2.0 Å and R-factors lower than 0.25 were selected for the PISCES dataset. The information of the two datasets are summarized in Table 1. Following the same procedure, the neural network models were trained with the VAST and PISCES data independently. The performance of each trained model was evaluated using the testing datasets from VAST and PISCES. As will be reported in the result section, two trained models have very similar performances on naturally occurred disulfide bond prediction. The neural network model trained with VAST data is used as the final model for engineered disulfide bond site prediction.

**Table 1:** The training and testing datasets from non-redundant databases.

	No. protein chains	No. of disulfide bonds	No. of negative data	Training Data	Testing Data
VAST	14,647	12,496	12,496	18,992	6,000
PISCES	15,139	5,122	5,122	8,244	2,000

### Data format of samples

Without considering hydrogen atoms, there are six atoms in each peptide bonded cysteine, namely, N, Cα, C, O, Cβ, and Sγ. To improve the robustness of the algorithm, the coordinate information of Sγ atoms was not used in the proposed algorithm. As a result, each pair of disulfide bonded cysteines can be represented using the coordinates of 10 atoms in the order of (N, Cα, C, O, Cβ, N’, Cα’, C’, O’, Cβ’), forming a 10 × 3 matrix. This coordinate matrix was converted to a distance matrix to remove the translation/rotation dependency, yielding a 10 × 10 Euclidean distance matrix. The same operation was used for the negative dataset. Because glycine does not have the required Cβ atom, it cannot be directly analyzed using this algorithm. The glycine should be mutated to alanine (or other type of amino acids) to predict the likelihood of forming engineered disulfide bond.

The averaged distance maps for the positive and negative sample sets reveal certain differences between positive and negative sample sets (Fig. 1b,c). These features are exploited by training the neural network model described in the next section to predict disulfide engineering sites.

### Neural network architecture

**Figure 2:**
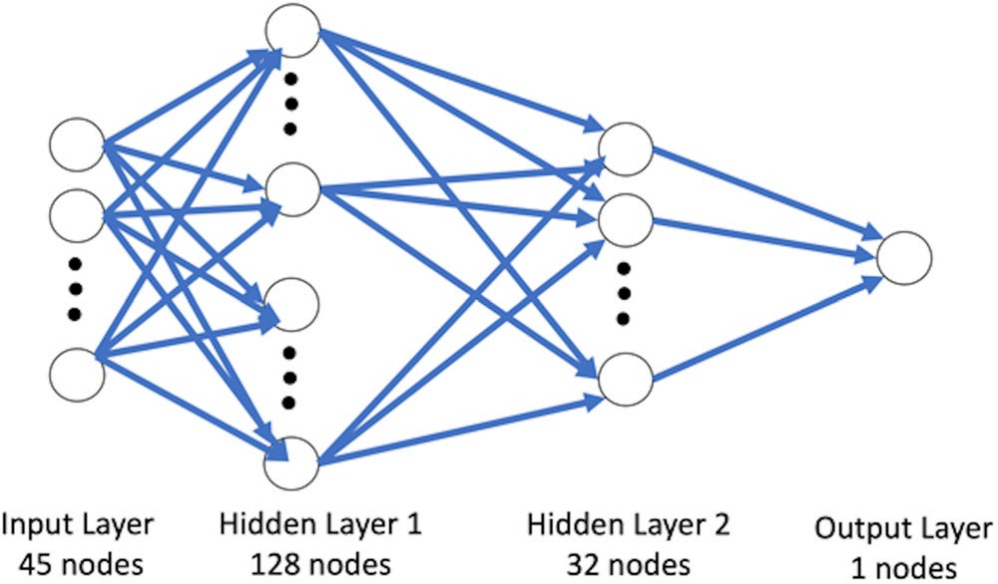
The fully connected neural network architecture. The input layer is the vector of 45 dimensions, followed by two hidden layers composed of nodes that can be activated based on the ReLu function.

A fully connected neural network was implemented and trained for classification to utilize pairwise atomic distance information. The overall architecture of the neural network is shown in Fig. 2. Because of the symmetry of the distance matrices and zero values of the diagonal elements, each matrix is reduced to a 45-dimensional vector. There are two hidden layers in the network, consisting of 128 and 32 units. In each hidden layer, a ReLu (Rectified Linear Units) operation was used as the activation function. Ten epochs were processed with the learning rate of 0.01 and a training batch size of 100 to obtain a converged trained network. The final output is a score between 0 and 1, and a threshold value (0.5) was used to classify the input as either bonded (1) or nonbonded (0).

### Prediction

**Figure 3:**
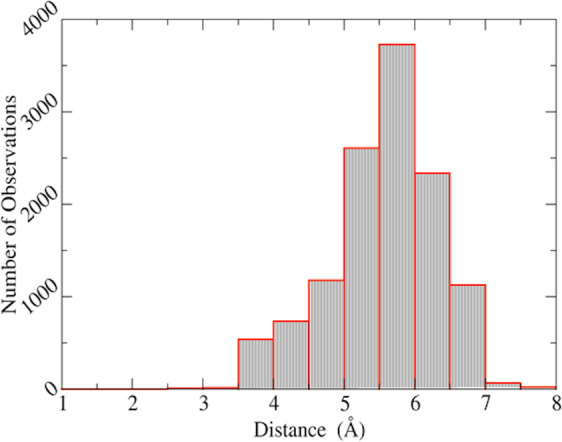
The histogram of distances between Cα atoms of disulfide bonded cysteines.

After training, the neural network model can be used to predict the formation of disulfide bonds between any pair of amino acids that can be mutated to cysteines (glycine residues need to be mutated to alanine before prediction). For each target protein with *n* residues, there will be *n(n-1)/2* pairs of residues that can potentially form disulfide bonds after cysteine substitution. It is possible to reduce the candidate list to those having higher probabilities of forming disulfide bonds using the distance between the Cα atoms of the considered residues as a prescreening mechanism. As shown in Fig. 3, the distances between the Cα atoms for the disulfide bonded cysteines are mainly between 3.0 Å and 7.5 Å. Using this criterion (distance between Cα atom in the range of 3.0 Å and 7.5 Å), the number of amino acid pairs is significantly reduced. For example, the Bril protein with 106 amino acids can potentially have 5,565 candidate residue pairs, and the prescreening distance criterion identifies that 378 of these residues are worthy of detailed examination.

### Performance evaluation

For the testing dataset extracted from naturally occurring disulfide bonds and the derived negative samples, the receiver operating characteristic (ROC) curve was used to assess the performance of the prediction algorithm. The sensitivity (i.e., true positive rate, TP/(TP + FN)) was plotted against the fall-out rate (i.e., false positive rate, FP/(FP + TN)) for the two testing datasets randomly selected from VAST or PISCES databases (see section 2.1). The area under the curve (AUC) was computed to evaluate the overall performance.

Besides the testing datasets, the algorithm was applied to engineered disulfide bond predictions. Three sets of data were used to assess the performance, specifically: (1) the set of 15 engineered disulfide bonds used in previous studies; (2) the alanine mutant models of 75 structures with disulfide bonds; and (3) experimentally tested 13 engineering sites (with both successful and failed engineered disulfide bonds) on Bril and Flavodoxin proteins. The results were compared with other methods.

## Results

### Performance on the testing dataset of natural disulfide bonds

**Figure 4:**
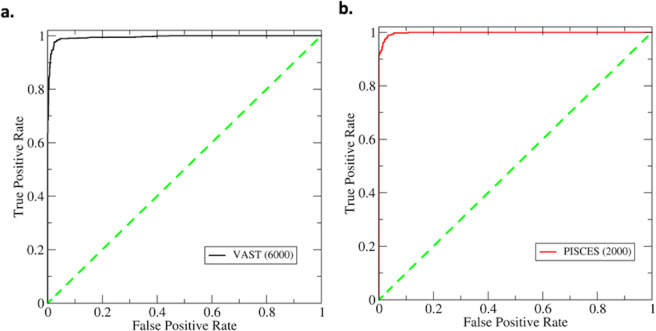
The classification performance using fully connected neural network model. (**a**) The receiver operating characteristic curve (ROC) on the VAST test dataset for the network trained with VAST data. (**b**) The ROC on the PISCES testing data for the same network. Both ROC curves show very good performance on the prediction of disulfide bonds. The number of the samples in each test dataset is shown in the parentheses. The area under the curve (AUC) are 0.995 for VAST test data and 0.998 for PISCES test data. The high true positive rate with extremely low false positive rate suggests the excellent performance in classifications.

Using the training datasets from VAST (18,992 samples), the neural network model was optimized to make predictions. First, the trained network was tested to evaluate the prediction performance using the dataset composed of 6,000 amino acid pairs in the VAST dataset. The receiver operating characteristic (ROC) curve is shown in Fig. 4a. The ROC curve shows that the high values of the true positive rates were achieved even with very low false positive rates. As a cross-validation, the same analysis was carried out for the PISCES testing dataset composed of 2,000 amino acid pairs (see Method section). Similar ROC curve was observed (Fig. 4b) for this PISCES dataset, indicating that the performance is not sensitive to the testing dataset. The area under the curve (AUC) are 0.995 and 0.998 for VAST and PISCES testing datasets respectively. When applying the algorithm in predicting potential engineering sites, the default cutoff value of 0.5 was used. The accuracy (defined as the correct prediction rate) is 0.99 for the classification positive samples (i.e., the native disulfide bonds) and the negative samples. This accuracy level means that both actual disulfide bonds and non-disulfide bonds (negative dataset) are correctly predicted. Assuming that the negative cases are trivial and the prediction accuracy is 100%, the positive cases must be 98% correct to have an overall 0.99 accuracy level, because the testing data is composed of equal number of data points.

### Prediction performance on engineered disulfide bonds

The ultimate goal of the algorithm is to predict the mutation sites that can eventually lead to the formation of engineered disulfide bonds after mutating the residues to cysteine. To evaluate the performance of the method, we first tested the prediction model with a published dataset composed of engineered disulfide bonds using their wild type structure as inputs^12^. There are 15 engineered disulfide bonds in the dataset, one of which involves mutation of glycine. The remaining 14 engineered disulfide bonds were all successfully predicted without modifying the PDB structures. The engineered disulfide bond by mutating glycine to cysteine (Gly79-Asn118 in the protein with PDBID 1SNO) was also correctly predicted by substituting the glycine with alanine by matching the main chain atoms of glycine and an alanine template generated from the VAST dataset. The performance was compared with the multiagent-based algorithm MAESTRO^16^ or the method combining geometry information with physical energy minimization/relaxation^12^ (see Table 2 for details). Although the overall ranking of the engineered disulfide bonds are not as good as that of other methods, the 100% success rate suggests the applicability of the proposed method in real applications. In another test, the performance was also compared with MAESTRO and method of Salam *et al*. using 75 proteins with disulfide bonds^12,16^. When using the original crystal structures as inputs, the proposed algorithm successfully predicted all 75 disulfide bonds (see supplementary materials). In a more elaborated test, these 75 protein structures were relaxed by minimizing the potential energies after mutating bonded cysteine to alanine. Then the relaxed structures were subjected to the disulfide bond mutation site prediction. We found that the performance of the *SSbondPre* on these alanine mutant structures was not as satisfactory. Out of 75 disulfide bonds in the relaxed structures, 71 were successfully predicted, yielding a success rate of 94.7%. This indicates that the neural network model can potentially benefit from an expanded training dataset by including the engineered disulfide bonds or even the precursors of those engineered disulfide bonds.

**Table 2:** The performance on the prediction of disulfide bond engineering sites.

PDB ID	Mutation	SSbondPre		MAESTRO		MAESTRO-Score		Salam *et al*.	
		Abs.Rank*	Rel.Rank**	Abs.Rank	Rel.Rank	Abs.Rank	Rel.Rank	Abs.Rank	Rel.Rank
1FG9	Glu7:A–Ser69:A	45	0.16	1	0.04	0	0	15	0.71
1LMB	Tyr88:3–Tyr88:4	2	0.03	10	0.2	9	0.18	32	0.48
1RNB	Ala43–Ser80	0	0.00	2	0.03	9	0.14	3	0.07
1RNB	Ser85–His102	15	0.41	33	0.52	40	0.63	0	0
1SNO	Gly79–Asn118***	45	0.73	3	0.04	4	0.05	22	0.34
1XNB	Ser100–Asn148	1	0.02	1	0.01	13	0.1	8	0.08
2CBA	Leu60–Ser173	33	0.33	66	0.45	68	0.46	55	0.47
2CI2	Thr22–Val82	9	0.60	5	0.18	4	0.14	0	0
2LZM	Ile9–Leu164	35	0.45	31	0.44	38	0.54	13	0.21
2RN2	Cys13–Asn44	5	0.12	14	0.16	21	0.24	25	0.33
2ST1	Thr22–Ser87	86	0.67	37	0.16	30	0.13	44	0.23
3GLY	Asn20–Ala27	207	0.95	104	0.39	163	0.62	187	0.82
3GLY	Thr246–Cys320	54	0.25	35	0.13	34	0.13	19	0.08
4DFR	Pro39–Cys85	35	0.45	11	*0.1*	11	*0.1*	16	*0.13*
9RAT	Ala4–Val118	35	0.65	8	0.15	10	0.19	25	0.68
Average		40.5	0.39	24.0	0.20	30.2	0.24	30.9	0.31
Median		35	0.41	14	0.16	21	0.18	19	0.23

^*^The absolute rank (abs. rank) was based on the scores of each method, starting with 0.

**The relative rank (rel. rank) was calculated as abs.rank/(total prediction-1).

***The glycine79 was substituted with alanine before *SSbondPre* prediction.

The performance on the prediction was further validated using two recently studied proteins that are frequently used as fusion partners in GPCR protein crystallization: Bril protein with 106 amino acids (PDB ID 1M6T), and Flavodoxin with 147 amino acids (PDB ID 1J8Q). The experiments were carried out in an independent study^13^, so the data can be considered as a double-blind test for the *SSbondPre* algorithm. For Bril, 10 mutants were tested in the experiment; 6 mutants formed disulfide bonds. In the case of Flavodoxin, three engineered mutants all formed disulfide bonds. The overall statistics are summarized in Table 3, and the detailed prediction results are listed in Table 4. The prediction outcome is good for both proteins, the top ranked predictions are more likely to form disulfide bonds. Using these 13 experimentally tested protein mutants as an independent testing data, the performance of the present method is evaluated and yields an accuracy of approximately 70% (9/13) (Table 4). The algorithm based on the support vector machine method with designed geometry features achieved 60% accuracy for the prediction of Bril disulfide bonds^13^. The multi-agent based method MaestroWeb correctly predicted 8 out of 13 engineered disulfide sites, and the DbD2 only correctly predicted 4 engineered sites for this dataset (see Table 4 for details). The poor performance of DbD2 might be due to the strict criteria in the default parameters of the DbD2 server, predicting only 23 and 21 engineering sites for Bril and Flavodoxin respectively (see Table 3).

**Table 3:** Summary for prediction results for Bril and Flavodoxin.

		Bril	Flavodoxin
Number of Candidate ssbonds^#^		378	464
Number of Predicted ssbonds	SSbondPre	40	54
	MaestroWeb	40	105
	DbD2	23	21
Experimentally tested mutants		10	3
Experimentally validated ssbonds		6	3

^#^Candidate bonds: number of amino acid pairs that passed the Cα distance criterion.

**Table 4:** Disulfide bond prediction results for the proteins Bril and Flavodoxin.

	Mutant	Formed disulfide bond^#^	Score*	*SSbondPre* (rank/total)	MaestroWeb(rank/total)	DbD2** (outcome)
Bril	Q41C-F65C	Yes	0.99	3/40	8/40	Yes
	A20C-Q25C	Yes	0.99	6/40	2/40	**No**
	T9C-A36C	Yes	0.99	7/40	13/40	Yes
	V16C-A29C	Yes	0.99	8/40	11/40	**No**
	L78C-A87C	Yes	0.99	9/40	9/40	Yes
	K27C-A79C	Yes	0.98	12/40	19/40	**No**
	*A75C-A90C*	*No*	*0.99*	*4/40*	*5/40*	*Yes*
	*A79C-A87C*	*No*	*0.98*	*10/40*	*10/40*	*Yes*
	*K51C-S55C*	*No*	*0.98*	*11/40*	*16/40*	*Yes*
	*S52C-S55C*	*No*	*0.84*	*28/40*	*27/40*	*Yes*
Flavodoxin	R125C-C102	Yes	0.99	5/54	3/105	Yes
	N14C-C93	Yes	0.97	13/54	15/105	No
	A43C-L74C	Yes	0.78	37/54	No	No

^#^Experimental observed bonds: Yes (bonded) or No (nonbonded).

*Score computed with the neural network model.

**DbD2 results are not ranked, so the outcomes are labelled as Yes (bonded) or No (nonbonded).

### Web server for disulfide bond prediction

**Figure 5:**
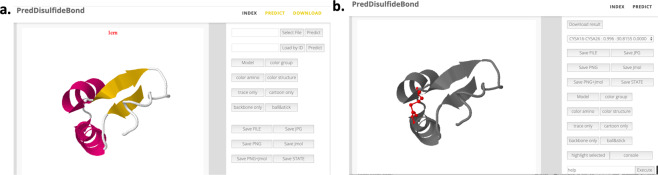
The web server for the prediction of disulfide bonds. (**a**) The input page for users to upload a PDB file or provide a PDB ID; (**b**) The result page with the selected prediction highlighted using the ball-stick representation and the backbone is shown in a cartoon representation.

A web server was established to provide access to the prediction algorithm. Users can either upload a PDB file or provide a PDB ID to use the prediction method (Fig. 5a). The prediction will be summarized as a list that can be pulled down to display the selected prediction sites, and the summary file can be downloaded in the Microsoft Excel format. The locations of the mutation sites are displayed and highlighted using the Jmol plugin^17^. In addition to the scores from the neural network model, the change in entropy due to the formation of engineered disulfide bond is also calculated using the empirical formula from Pace *et al*.^18^. The impact of the mutation to the energy change relative to the wild type protein was evaluated using a statistical potential energy derived from the neighborhood preference of each amino acid^19^. An illustrative example of the prediction results from the web server is shown in Fig. 5b. If a batch mode prediction is desired, the command line program is recommended. The program code is implemented in Python and is available at the Github repository (https://github.com/LiuLab-CSRC/SSBONDPredict).

## Discussions and Conclusion

Structural-based disulfide design has been successful in many cases. It is even possible to cross-link with disulfide bond(s) between two different domains to enhance stability^20^. The structural features selected based on experience or knowledge about disulfide bonds have been demonstrated to be successful to a good extent, yet the existing methods mainly focus on limited selection of features, such as distance, angle, and torsion angles formed by the atoms surrounding the S-S bonds. Here, we attempt to think out of the box by utilizing machine learning methods to extract important features automatically. Disulfide bonds were represented in their most original form with atomic coordinates, without defining derived parameters. The transformation from the Cartesian coordinates to internal coordinates facilitates the feature extraction for machine learning methods by removing the translational and rotational dependencies.

**Figure 6:**
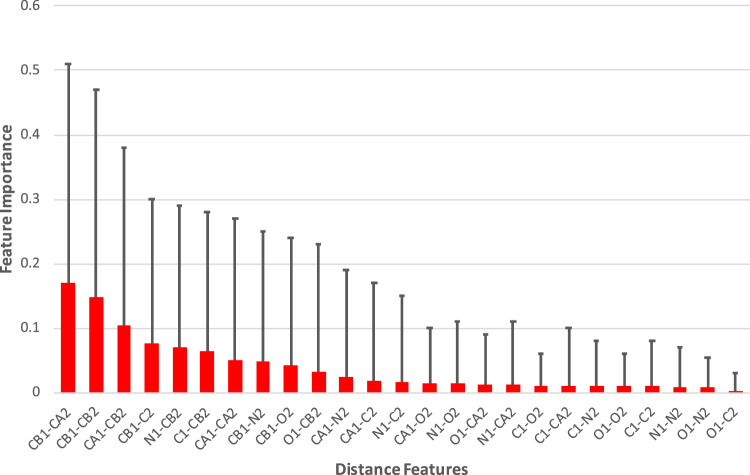
The relevance of the distance features to the classification outcome. Out of the 45 unique distances, 20 distances have negligible influence on the classification performance. The distances between Cβ and main chain atoms of the pairing residue are important features in disulfide bond classifications.

The distance matrix was reduced to 45 unique variables by removing symmetric items and diagonal entries. We studied the relevance of the distance features using the random forest regression method. It is clear that the atomic distances between residues are the essential features for correct classification, while the distances between atoms of the same residue have neglectable impacts on the classification accuracy. Nonetheless, the positions of 10 atoms are essential for the algorithm, as shown in Fig. 6: the 10 most important distance features involve all 10 atoms from both residues. In most existing algorithms, only the features involving the connected atoms of Cα-Cβ-Sγ-Sγ’-Cβ’-Cα’ are utilized, the method presented in this study show that the main chain atoms (C,N, O, C’, N’,O’) are also very important. For example, the distances between Cβ and the main chain atoms (C,N,O) of the other bonding residue have significant weights, revealed by the regression test. The incorporation of these additional features should improve the prediction accuracy. Furthermore, we did not use the information for the sulfur atoms (Sγ or Sγ’) because (1) the coordinates for sulfur atoms are not available if point mutation is required; and (2) the distance between sulfur atoms will dominate the classifier.

For supervised classification using machine learning methods, the selection of dataset is crucial. The training of neural network model using the labelled datasets is to optimize the hyperplanes that enclose the positive samples as closely as possible. The positive samples, the disulfide bonded cysteine pairs in this study, are clearly defined and readily extracted using the computer programs from protein structures. The selected negative samples are a subset of the mutually exclusive set where the amino acid pairs are not disulfide bonded. The procedure described in the method section identified a subset of amino acid pairs that are spatially close, and not disulfide bonded. Furthermore, these amino acids adjacent to the bonded cysteines are also not optimal mutation sites, as consecutive disulfide bonds are very unusual (the analysis on high resolution PDB structures revealed 33 pairs of adjacent disulfide bonds out of 100,271 disulfide bonds). The basic principle for the selection of negative dataset is to form training datasets that well define the hyperplanes enclosing the disulfide bonded cysteines (Figure S1). Other negative datasets may achieve the same goal, with variations in the hyperplanes, which affect the prediction powers. The excellent performance on the identification of the naturally occurring disulfide bonds in the present work suggests that the training datasets enabled the feature learning by the neural network, without using the coordinate information of sulfur atoms (see Figure S2).

Although the presented algorithm did not explicitly address conformational changes induced by point mutations, the success in the prediction of the mutation sites to form engineered disulfide bonds suggested that the method has merit in protein engineering applications. The coordinate information of sulfur atoms was omitted in the prediction algorithm intentionally, leaving a large degree of freedom for conformational changes in the predicted bonded residues. The starting structure does not have to be an experimentally determined model as it has been shown that high quality in silico predicted models can serve as a starting model^13^. As shown in the case of relaxed structures with alanine mutants, the proposed algorithm can predict about 95% mutation sites. The reduced accuracy suggests that the trained neural network model still has room for improvement. One project for future research will be training the neural network model with an expanded dataset that includes relaxed structures of alanine mutants (or mutants of other amino acids) as positive samples. On the other hand, the lower accuracy for alanine mutants compared to the wild type suggests a potential improvement of accuracy for cysteine mutants by combining point mutation and structure refinement methods. The structure refinement often requires extensive computational modeling, making it impractical for all mutants (hundreds or thousands candidates even after applying the Cα distance filtering as described in this work). The advantage of this method is the high throughput of the prediction, taking only seconds for typical sized proteins. The output can provide candidate mutants for disulfide bond engineering. For those mutants with special interest, it is worthwhile to construct cysteine mutant and refine the structure prior to the prediction.

In summary, the machine learning method was implemented to predict the sites for disulfide bond engineering using ‘learned’ features. The testing results using natural disulfide bonds show that the method can achieve high accuracy levels. For engineered disulfide bond prediction, the accuracy level is around 70%, making it useful in guiding the disulfide bond engineering. The program and the associated web server are available at http://liulab.csrc.ac.cn/ssbondpre/.

## Supplemenatry information

Supplemenatry information.
