# Author Correction: Prediction of disulfide bond engineering sites using a machine learning method

**DOI:** 10.1038/s41598-020-69841-y

**Published:** 2020-07-28

**Authors:** Xiang Gao, Xiaoqun Dong, Xuanxuan Li, Zhijie Liu, Haiguang Liu

**Affiliations:** 1 Complex Systems Division, Beijing Computational Science Research Center, 8 E Xibeiwang Rd, Haidian, Beijing, 100193 People’s Republic of China; 2 School of Software Engineering, University of Science and Technology China, Suzhou, Jiang Su 215123 People’s Republic of China; 3 Department of Engineering Physics, Tsinghua University, Haidian, Beijing, 100084 People’s Republic of China; 4 iHuman Institute, ShanghaiTech University, 393 Middle Huaxia Rd, Pudong, Shanghai, 201210 People’s Republic of China

Correction to: *Scientific Reports* https://doi.org/10.1038/s41598-020-67230-z, published online 25 June 2020

This Article contains typographical errors in the Acknowledgements section.

“The funding from National Natural Science Foundation of China is acknowledged, grant numbers: 31971136, U1530402, U1430237.”

should read:

“The funding from National Natural Science Foundation of China is acknowledged, grant numbers: 31971136, U1930402, U1430237.”

