# Supplementary material for: Prediction of disulfide bond engineering sites using a machine learning method
Source: Sci Rep. 2020 Jun 25;10:10330. doi: 10.1038/s41598-020-67230-z (PMC7316719; doi:10.1038/s41598-020-67230-z)
Supplement: Supplementary file 1 — Supplemenatry information. [file 41598_2020_67230_MOESM1_ESM.pdf]

Prediction of disulfide bond engineering sites using a machine learning method

Xiang Gao[ab], Xiaoqun Dong[ab], Xuanxuan Li[ca], Zhijie Liu[d] and Haiguang Liu[a]*
[a]Complex Systems Division, Beijing Computational Science Research Center, 8 E Xibeiwang Rd, Haidian, Beijing, 100193, People's Republic of China
[b]School of Software Engineering, University of Science and Technology China, Suzhou, Jiang Su, 215123, People's Republic of China
[c]Department of engineering physics, Tsinghua University, Haidian, Beijing, 100084, People's Republic of China
[d]iHuman Institute, ShanghaiTech University, 393 Middle Huaxia Rd, Pudong, Shanghai, 201210, People's Republic of China
Correspondence email: hgliu@csrc.ac.cn

Supporting information

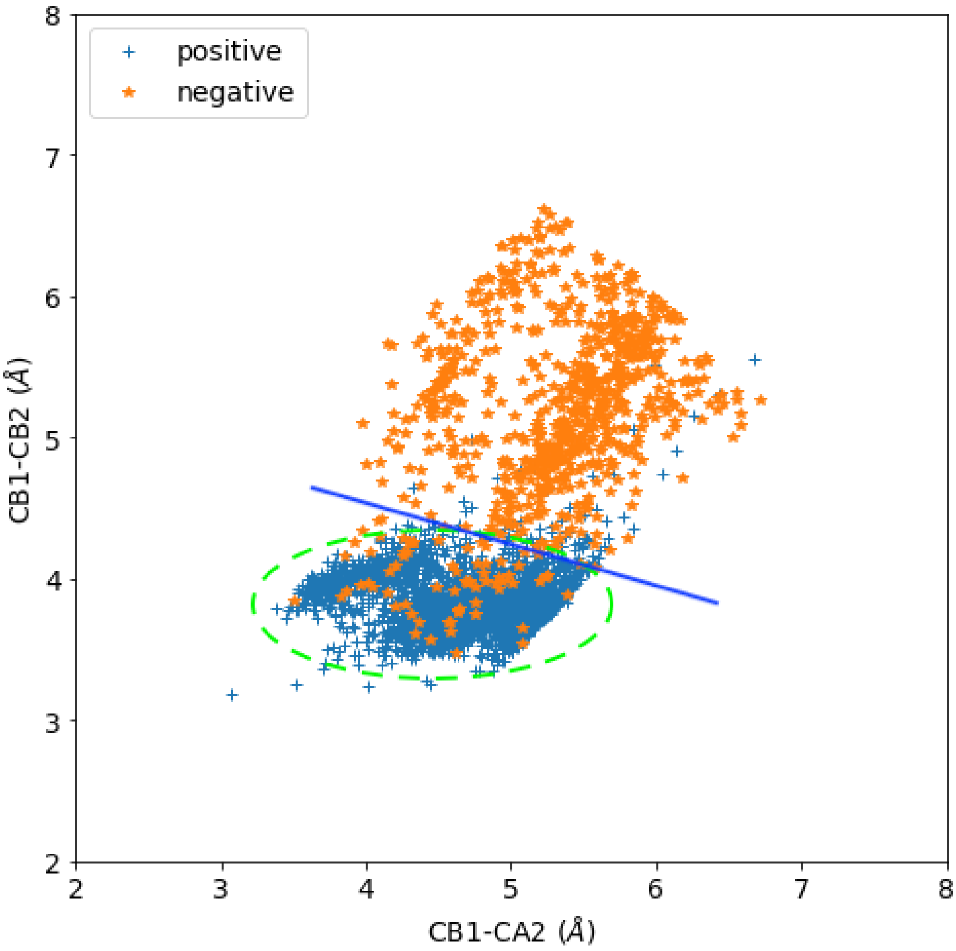

Figure S1. The positive/negative data points in the feature space spanned by two inter-atom distances (CB1-CA2, CB1-CB2). The positive dataset (blue crosses) is composed of cysteines with naturally occurred disulfide bonds. The negative dataset (orange stars) helps define the hyperplane that separates the positive and negative data points. The false positives (orange stars in the green circle) indicates that a linear classifier (the blue line indicates the boundary) is not sufficient to accurately classify the data.

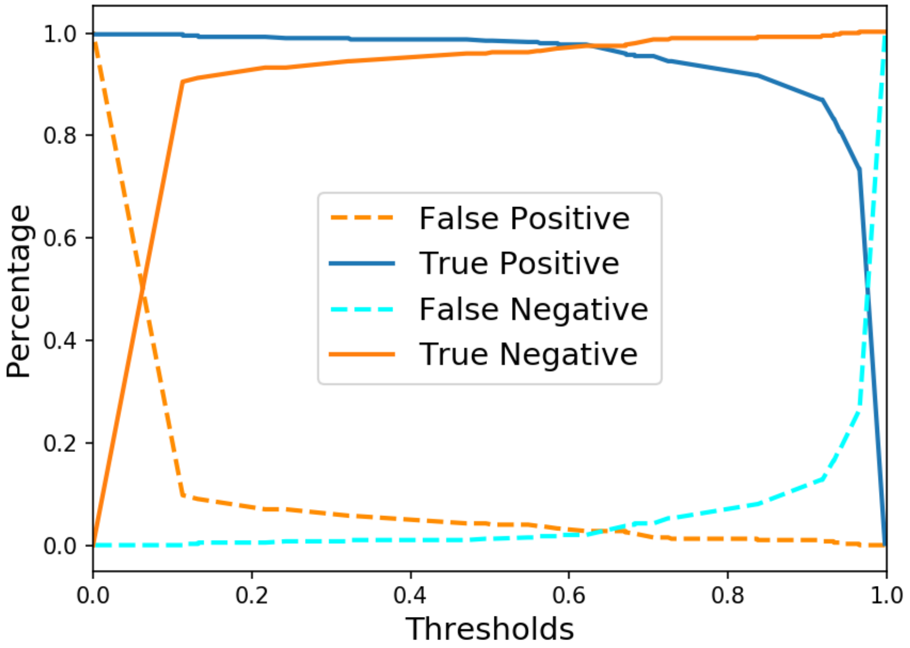

Figure S2. The performance of the machine learning classification algorithm at various threshold levels. At the default threshold 0.5, the true positive rate = 0.987.

Table S1. The performance on 75 natural disulfide bonds in wild type proteins.

			**SSbondPre**			**Maestro**		**Maestro-Score**		**Salam et al.**
**PDB ID**	resid 1	resid 2	score	Abs. rank	Rel. rank	Abs. Rank	Rel. Rank	Abs. Rank	Rel. Rank	Rel. Rank
**1aba**	14	17	0.901	3	0.17	1	0.03	4	0.10	0.00
**1c7k**	99	112	0.991	3	0.07	1	0.02	8	0.12	0.00
**1dyq**	96	106	0.977	13	0.16	16	0.12	23	0.18	0.05
**1gv9**	198	238	0.925	29	0.36	14	0.09	33	0.21	0.04
**1kng**	92	95	0.897	14	0.38	6	0.09	12	0.18	0.02
**1lf7**	76	168	0.993	3	0.06	4	0.04	8	0.08	0.07
**1lju**	82	89	0.995	2	0.05	1	0.02	1	0.02	0.17
**1m40**	77	123	0.994	7	0.06	13	0.08	27	0.17	0.17
**1mf7**	128	318	0.983	7	0.10	7	0.06	20	0.18	0.09
**1mjn**	161	299	0.991	5	0.08	5	0.05	11	0.11	0.03
**1nko**	46	106	0.532	41	1.00	6	0.09	6	0.09	0.02
**1oal**	52	147	0.990	2	0.05	3	0.03	0	0.00	0.03
**1olr**	6	35	0.966	10	0.14	23	0.15	41	0.26	0.00
**1p3c**	32	48	0.995	2	0.02	12	0.08	26	0.17	0.06
**1qgv**	38	79	0.980	5	0.12	6	0.10	5	0.08	0.35
**1qk8**	40	43	0.910	12	0.26	0	0.00	0	0.00	0.02
**1r26**	30	33	0.858	13	0.45	3	0.06	3	0.06	0.05

**1rie**	144	160	0.999	1	0.02	11	0.15	22	0.30	0.62
**1shu**	39	218	0.992	4	0.07	18	0.19	23	0.24	0.01
**1t2i**	7	96	0.606	27	0.84	7	0.17	13	0.31	0.00
**1t2j**	22	92	0.998	1	0.02	11	0.13	18	0.22	0.09
**1unr**	60	77	0.975	4	0.13	14	0.30	7	0.15	0.06
**1vhu**	111	154	0.980	12	0.15	17	0.14	14	0.12	0.02
**1wcu**	63	141	0.991	3	0.06	5	0.05	15	0.15	0.10
**1xbu**	245	250	0.778	67	0.60	9	0.05	29	0.16	0.07
**1xt5**	26	109	0.997	2	0.04	7	0.08	11	0.13	0.05
**1y9l**	69	95	0.994	1	0.03	9	0.17	15	0.28	0.00
**1zk5**	53	110	0.940	15	0.31	29	0.27	36	0.34	0.00
**2a6y**	151	185	0.955	19	0.24	14	0.10	22	0.16	0.10
**2a6z**	151	185	0.952	21	0.27	10	0.08	23	0.18	0.05
**2aqm**	55	150	0.992	3	0.07	15	0.15	15	0.15	0.01
**2ce0**	67	73	0.994	1	0.04	0	0.00	1	0.02	0.18
**2e0q**	64	67	0.873	14	0.54	3	0.07	6	0.13	0.02
**2erf**	153	214	0.991	4	0.06	2	0.01	2	0.01	0.09
**2fwg**	461	464	0.890	14	0.47	0	0.00	1	0.02	0.05
**2hsh**	32	35	0.913	13	0.36	5	0.11	6	0.13	0.03
**2i1u**	37	40	0.896	17	0.52	3	0.06	2	0.04	0.00
**2i4a**	32	35	0.889	13	0.41	0	0.00	6	0.13	0.03
**2icc**	22	94	0.997	1	0.03	5	0.10	4	0.08	0.00
**2nwf**	134	151	0.998	1	0.02	7	0.07	25	0.26	0.15
**2p39**	95	113	0.913	18	0.32	34	0.44	34	0.44	0.07
**2p52**	173	239	0.997	1	0.02	4	0.04	12	0.12	0.03
**2py0**	129	142	0.921	13	0.30	9	0.15	9	0.15	0.07
**2qo4**	80	91	0.982	7	0.14	0	0.00	1	0.02	0.07
**2rkq**	48	54	0.993	1	0.02	11	0.10	23	0.21	0.01
**2vyo**	22	215	0.784	43	0.57	10	0.10	24	0.24	0.01
**2xfd**	90	101	0.995	2	0.05	1	0.01	7	0.10	0.00
**2yxf**	25	80	0.998	1	0.05	0	0.00	0	0.00	0.06
**3cb9**	147	204	0.995	1	0.01	4	0.03	11	0.08	0.04
**3e8t**	8	15	0.990	2	0.03	1	0.01	4	0.04	0.03
**3edi**	42	198	0.997	1	0.01	4	0.04	8	0.08	0.01
**3fsa**	3	26	0.997	2	0.04	0	0.00	5	0.07	0.00
**3fz4**	10	13	0.888	16	0.46	3	0.06	4	0.08	0.05
**3ga4**	55	58	0.912	12	0.27	3	0.05	4	0.07	0.02
**3gnz**	37	63	0.781	47	0.62	7	0.06	22	0.19	0.00
**3gui**	21	142	0.990	5	0.08	3	0.05	1	0.02	0.04
**3hnb**	2174	2326	0.985	5	0.08	5	0.05	14	0.15	0.03
**3hz8**	57	60	0.878	40	0.62	8	0.09	12	0.13	0.04
**3kff**	64	157	0.996	1	0.02	0	0.00	0	0.00	0.05

**3l4r**	64	157	0.996	1	0.02	5	0.06	7	0.08	0.00
**3m1w**	5	64	0.974	12	0.12	0	0.00	10	0.07	0.07
**3o22**	89	186	0.996	1	0.02	0	0.00	2	0.02	0.03
**3rt2**	27	153	0.963	12	0.16	10	0.11	25	0.28	0.06
**3rxw**	68	237	0.996	2	0.02	46	0.29	67	0.42	0.02
**3seb**	93	113	0.997	1	0.01	0	0.00	1	0.01	0.19
**3sh4**	159	193	0.995	1	0.02	3	0.02	6	0.04	0.04
**3t0v**	23	88	0.997	1	0.03	15	0.21	16	0.22	0.16
**3tpk**	22	96	0.996	1	0.03	8	0.11	9	0.13	0.00
**3vor**	106	170	0.995	2	0.03	6	0.05	20	0.18	0.05
**3zyp**	22	52	0.694	53	0.74	8	0.06	35	0.25	0.02
**4eq8**	7	148	0.995	4	0.07	4	0.04	12	0.12	0.03
**4f0w**	7	148	0.997	1	0.02	3	0.03	14	0.14	0.00
**4fh4**	77	123	0.993	6	0.05	6	0.04	14	0.08	0.04
**4ftf**	74	111	0.995	1	0.03	0	0.00	0	0.00	0.00
**4hwm**	68	124	0.992	1	0.03	0	0.00	1	0.02	0.00
Mean			**0.947**	**9.96**	**0.19**	**7.24**	**0.08**	**13.04**	**0.13**	**0.06**

Table S2. The performance on alanine mutants of 75 natural disulfide bonds.

			**SSbondPre**			**Maestro**		**Maestro-Score**		**Salam et al.**
**PDB ID**	Resid 1	Resid 2	score	Abs. Rank	Rel. Rank	Abs. Rank	Rel. Rank	Abs. Rank	Rel. Rank	Rel. Rank
**1aba**	14	17	0.831	12	0.43	0	0.00	1	0.02	0.00
**1c7k**	99	112	0.973	5	0.09	3	0.04	12	0.17	0.00
**1dyq**	96	106	0.985	11	0.10	5	0.04	12	0.09	0.05
**1gv9**	198	238	0.516	104	0.97	103	0.63	125	0.76	0.04
**1kng**	92	95	0.774	35	0.69	23	0.29	39	0.49	0.02
**1lf7**	76	168	0.996	1	0.02	1	0.01	3	0.03	0.07
**1lju**	82	89	0.994	1	0.01	3	0.04	2	0.03	0.17
**1m40**	77	123	0.972	36	0.24	50	0.34	91	0.63	0.17
**1mf7**	128	318	0.612	75	0.82	-	-	-	-	0.09
**1mjn**	161	299	0.955	28	0.31	37	0.36	45	0.44	0.03
**1nko**	46	106	-	-	-	9	0.13	15	0.22	0.02
**1oal**	52	147	0.987	1	0.02	2	0.02	4	0.04	0.03
**1olr**	6	35	0.966	12	0.13	27	0.17	44	0.28	0.00
**1p3c**	32	48	0.99	6	0.05	22	0.14	42	0.26	0.06
**1qgv**	38	79	0.98	4	0.07	2	0.03	3	0.05	0.35
**1qk8**	40	43	0.858	19	0.32	0	0.00	4	0.06	0.02
**1r26**	30	33	0.801	21	0.60	4	0.08	6	0.12	0.05
**1rie**	144	160	0.99	5	0.09	12	0.16	24	0.32	0.62
**1shu**	39	218	0.996	0	0.00	9	0.08	15	0.13	0.01

**1t2i**	7	96	0.619	37	0.86	5	0.11	10	0.23	0.00
**1t2j**	22	92	0.998	0	0.00	24	0.31	23	0.30	0.09
**1unr**	60	77	0.739	23	0.59	-	-	-	-	0.06
**1vhu**	111	154	0.933	31	0.29	21	0.17	21	0.17	0.02
**1wcu**	63	141	0.975	14	0.22	9	0.08	19	0.18	0.10
**1xbu**	245	250	0.888	68	0.48	8	0.05	20	0.11	0.07
**1xt5**	26	109	0.998	0	0.00	13	0.15	13	0.15	0.05
**1y9l**	69	95	0.99	0	0.00	12	0.25	20	0.42	0.00
**1zk5**	53	110	0.969	7	0.12	17	0.15	27	0.25	0.00
**2a6y**	151	185	0.958	21	0.19	4	0.03	17	0.12	0.10
**2a6z**	151	185	0.957	23	0.24	9	0.06	24	0.17	0.05
**2ce0**	67	73	0.995	0	0.00	0	0.00	0	0.00	0.01
**2e0q**	64	67	0.839	19	0.49	2	0.04	6	0.12	0.18
**2erf**	153	214	0.981	10	0.10	9	0.06	10	0.07	0.02
**2fwg**	461	464	0.679	32	0.68	8	0.16	12	0.24	0.09
**2hsh**	32	35	0.805	25	0.50	2	0.04	4	0.07	0.05
**2i1u**	37	40	0.881	23	0.50	5	0.09	12	0.21	0.03
**2i4a**	32	35	0.83	20	0.44	4	0.07	5	0.09	0.00
**2icc**	22	94	0.993	0	0.00	25	0.45	26	0.46	0.03
**2nwf**	134	151	0.989	5	0.08	18	0.20	37	0.42	0.00
**2p39**	95	113	0.935	11	0.16	21	0.26	24	0.29	0.15
**2p52**	173	239	0.987	8	0.11	6	0.06	21	0.20	0.07
**2py0**	129	142	0.637	47	0.85	46	0.79	45	0.78	0.03
**2qo4**	80	91	0.993	2	0.04	0	0.00	0	0.00	0.07
**2rkq**	48	54	0.992	2	0.03	11	0.11	25	0.24	0.07
**2vyo**	22	215	0.641	70	0.76	14	0.15	24	0.26	0.01
**2xfd**	90	101	0.981	7	0.15	4	0.06	9	0.13	0.01
**2yxf**	25	80	0.998	0	0.00	0	0.00	0	0.00	0.00
**3cb9**	147	204	-	-	-	13	0.10	44	0.33	0.06
**3e8t**	8	15	0.99	4	0.05	4	0.03	20	0.17	0.04
**3edi**	42	198	-	-	-	1	0.01	5	0.04	0.03
**3fsa**	3	26	0.997	1	0.01	2	0.03	12	0.15	0.01
**3fz4**	10	13	0.86	18	0.41	2	0.04	4	0.07	0.00
**3ga4**	55	58	0.672	51	0.73	10	0.16	21	0.33	0.05
**3gnz**	37	63	0.714	75	0.70	1	0.01	18	0.14	0.02
**3gui**	21	142	0.987	8	0.09	8	0.11	8	0.11	0.00
**3hnb**	2174	2326	0.993	2	0.03	14	0.13	30	0.28	0.04
**3hz8**	57	60	0.807	49	0.62	5	0.06	10	0.11	0.03
**3kff**	64	157	0.988	7	0.11	2	0.03	4	0.05	0.04
**3l4r**	64	157	0.995	1	0.01	6	0.06	9	0.10	0.05
**3m1w**	5	64	0.963	18	0.16	4	0.03	13	0.08	0.00
**3o22**	89	186	0.997	0	0.00	10	0.10	13	0.14	0.07
**3rt2**	27	153	0.961	15	0.17	39	0.43	62	0.69	0.03
**3rxw**	68	237	0.998	0	0.00	7	0.04	23	0.14	0.06

**3seb**	93	113	0.989	12	0.11	4	0.03	20	0.16	0.02
**3sh4**	159	193	0.997	1	0.01	13	0.09	20	0.14	0.19
**3t0v**	23	88	0.998	0	0.00	25	0.32	27	0.34	0.04
**3tpk**	22	96	0.996	0	0.00	13	0.16	13	0.16	0.16
**3vor**	106	170	0.995	2	0.02	21	0.17	26	0.21	0.00
**3zyp**	22	52	-	-	-	69	0.46	116	0.78	0.05
**4eq8**	7	148	0.996	0	0.00	5	0.05	13	0.13	0.02
**4f0w**	7	148	0.995	2	0.03	8	0.08	12	0.12	0.03
**4fh4**	77	123	0.957	40	0.26	80	0.47	115	0.68	0.00
**4ftf**	74	111	0.991	1	0.03	0	0.00	2	0.05	0.04
**4hwm**	68	124	0.994	1	0.02	3	0.04	5	0.07	0.00
**4l05**	55	150	0.989	2	0.03	4	0.04	8	0.07	0.00
Mean			**0.92**	**16.8**	**0.23***	**13.5**	**0.13**	**22.1**	**0.21**	**0.06**

The training/testing dataset with non-redundancy filtering is available as supporting data, available in the Github repositoary at: https://github.com/LiuLab-CSRC/SSBONDPredict/tree/master/Dataset .

The machine learning based method, *SSbondPre*, was tested using a set of structures with naturally occurring disulfide bonds. The structures were obtained from the Maestro website[1]. In the second test, bonded cysteine residues were mutated to alanine, then the structures were subjected to energy minimization to relax the geometry. The mutant structures were analyzed using the proposed method to predict the engineering sites to form disulfide bonds. The results are summarized in Table S1 and S2 for the wild type proteins and the alanine mutants. The prediction results for Maestro, Maestro-Score, and the method by Salam et al.[2] were obtained from the Laimer et al.[3].

Note:
Abs. rank: Absolutely rank starts from 0.
Rel. rank: calculated as Abs.Rank/(Number of predicted bonds-1).

The mean values were calculated for successfully predicted disulfide bonds. If the failed cases were assigned to have relative rank of 1.0, then *SSbondPre* has a mean value of 0.27 for the relative rank.

Reference

1. Laimer, J., Hiebl-Flach, J., Lengauer, D. & Lackner, P. MAESTROweb: A web server for structure-based protein stability prediction. *Bioinformatics* **32**, 1414–1416 (2016).
2. Salam, N. K., Adzhigirey, M., Sherman, W., Pearlman, D. A. & Thirumalai, D. Structure-based approach to the prediction of disulfide bonds in proteins. in *Protein Engineering, Design and Selection* **27**, 365–374 (Oxford University Press, 2014).
3. Laimer, J., Hofer, H., Fritz, M., Wegenkittl, S. & Lackner, P. MAESTRO - multi agent stability prediction upon point mutations. *BMC Bioinformatics* **16**, 1966–1971 (2015).
